# Application of Mesenchymal Stem Cells in Targeted Delivery to the Brain: Potential and Challenges of the Extracellular Vesicle-Based Approach for Brain Tumor Treatment

**DOI:** 10.3390/ijms222011187

**Published:** 2021-10-17

**Authors:** Anh Duy Do, Ida Kurniawati, Chia-Ling Hsieh, Tai-Tong Wong, Yu-Ling Lin, Shian-Ying Sung

**Affiliations:** 1 International Ph.D. Program for Translational Science, College of Medical Science and Technology, Taipei Medical University, Taipei 110, Taiwan; d623109002@tmu.edu.tw (A.D.D.); chsieh0404@gmail.com (C.-L.H.); 2 Department of Physiology, Pathophysiology and Immunology, Pham Ngoc Thach University of Medicine, Ho Chi Minh City 700000, Vietnam; 3 The Ph.D. Program for Translational Medicine, College of Medical Science and Technology, Taipei Medical University, Taipei 110, Taiwan; m610108011@tmu.edu.tw; 4 Graduate Institute of Clinical Medicine, College of Medicine, Taipei Medical University, Taipei 110, Taiwan; ttwong99@gmail.com; 5 Pediatric Brain Tumor Program, Taipei Cancer Center, Taipei Medical University, Taipei 110, Taiwan; 6 Neuroscience Research Center, Taipei Medical University Hospital, Taipei 110, Taiwan; 7 Division of Pediatric Neurosurgery, Department of Neurosurgery, Taipei Medical University Hospital and Taipei Neuroscience Institute, Taipei Medical University, Taipei 110, Taiwan; 8 Agricultural Biotechnology Research Center, Academia Sinica, Taipei 115, Taiwan; lyring41@gmail.com; 9 TMU Research Center of Cancer Translational Medicine, Taipei Medical University, Taipei 110, Taiwan; 10 Office of Human Research, Taipei Medical University, Taipei 110, Taiwan; 11 TMU-Research Center of Urology and Kidney, Taipei Medical University, Taipei 110, Taiwan

**Keywords:** mesenchymal stem cell, extracellular vesicle, exosome, brain tumor, blood-brain barrier, targeted delivery, gene delivery, cell-based therapy, cell-free therapy

## Abstract

Treating brain tumors presents enormous challenges, and there are still poor prognoses in both adults and children. Application of novel targets and potential drugs is hindered by the function of the blood-brain barrier, which significantly restricts therapeutic access to the tumor. Mesenchymal stem cells (MSCs) can cross biological barriers, migrate to sites of injuries to exert many healing effects, and be engineered to incorporate different types of cargo, making them an ideal vehicle to transport anti-tumor agents to the central nervous system. Extracellular vesicles (EVs) produced by MSCs (MSC-EVs) have valuable innate properties from parent cells, and are being exploited as cell-free treatments for many neurological diseases. Compared to using MSCs, targeted delivery via MSC-EVs has a better pharmacokinetic profile, yet avoids many critical issues of cell-based systems. As the field of MSC therapeutic applications is quickly expanding, this article aims to give an overall picture for one direction of EV-based targeting of brain tumors, with updates on available techniques, outcomes of experimental models, and critical challenges of this concept.

## 1. Introduction

Brain tumors are widely known as a dismal category of cancers in both adults and children. They can be fast-developing and located in critical brain regions, leading to ineffective treatment and exceptionally poor prognoses. The most common type of malignant central nervous system (CNS) tumor in adults is glioblastoma multiforme (GBM), the most prevalent and also the most aggressive form of glioma. GBM accounts for 14.5% of all CNS tumors and 48.6% of all primary malignant CNS tumors [1]. Although only 2% of primary neoplasms occur in the CNS, such cases account for 7% of all cancer-related deaths [2]. More than two-thirds of GBM patients die within 2 years after diagnosis with a median survival time of only 8 months [2,3]. The 5-year survival rate in patients receiving standard-of-care treatment is less than 10%, with a recurrence rate of 90% [4]. In pediatric populations, brain neoplasms are not only the most common type of solid tumor [2], but also the leading cause of childhood cancer mortality, with a 5-year survival rate of less than 5% for GBM [5].

Current standard treatments of GBM are a combination of surgical resection, radiation, and chemotherapy [6]. Due to diffuse infiltration of these tumors, complete resection is challenging and rarely achieved. Adjuvant radiotherapy is often required to eliminate remaining cancerous tissues, but this also presents with severe complications such as hormonal dysfunctions, cognitive impairments and neurological disorders [7]. Exposure of the developing brain to radiotherapy also causes irreversible developmental sequelae in surviving children when they enter adulthood [8]. Chemotherapy is applied as primary or adjuvant treatment for brain tumors, but presents issues of inadequate drug delivery to the CNS, systemic toxicities and drug resistance. Therefore, the idea of targeted cancer therapy, which aims to precisely attack cancer cells but spares vulnerable normal tissues, is attractive and especially relevant in the case of brain tumors.

Increasing numbers of druggable targets for brain tumors are being discovered, while preclinical studies have proposed many candidate drugs or genes with robust anti-cancer properties [9]. However, most of them have exhibited disappointing therapeutic performance in vivo due to poor bioavailability and adverse effects on healthy tissues [10]. One major obstacle in the medical treatment of brain disorders is the blood-brain barrier (BBB), which maintains strict homeostasis of the CNS’s privileged microenvironment and protects neural cells from offensive mediators in the blood. As one of the most complex and selective biological barriers in human body, the BBB also impedes penetration of most therapeutic drugs and thus prevents their accumulation to a desirable concentration in tumors [11]. The vicious dilemma of higher dosages needed for therapeutic effects on the tumor and lower dosages needed for fewer side effects on healthy tissues has hindered translation of many potential agents.

Stem cell-based delivery systems have been established on the notion that transplanted stem cells can actively penetrate the BBB and migrate to tumor site [12,13,14], making them an ideal carrier for anti-cancer agents [15]. Among potential cells sources, mesenchymal stem cells (MSCs) are often highlighted as having greater availability, ease of expansion ex vivo, and low immunogenicity in vivo. Meanwhile, stem cells were proven to secrete extracellular vesicles (EVs) as key effectors for most of their therapeutic actions [16,17]. EVs are natural and generally inherit many features of their parent cells. There exists a strategic shortcut in which therapeutic MSCs can be replaced by their EVs to achieve many of the same desirable effects. The small size, endogenous origin and readiness for modification are unique advantages of EVs, which help them maintain in vivo biostability and effective targeted drug delivery to the brain [18,19].

In this review, we provide a brief introduction to cancer therapies based on MSCs and their critical issues, then give particular attention to the therapeutic potential of MSC-derived EVs, their applicable techniques, research status, and current challenges in brain tumor treatment.

## 2. Mesenchymal Stem Cells (MSCs) and Their Therapeutic Potential for Brain Diseases

### 2.1. Cell Origins and Therapeutic Functions

MSCs are a heterogeneous cell population, with about 1 per 10,000 nucleated cells present after birth and gradually diminishing in number as a result of aging [20]. MSCs are multipotent with natural differentiation into cells of mesodermal origin and possible transdifferentiation into ectodermal or endodermal lineages [21]. In addition to those found in bone marrow (BM-MSCs), MSCs can be isolated from various sources in the body such as adipose tissue (AT-MSCs), umbilical cord (UC-MSCs), Wharton’s jelly (WJ-MSCs), placenta, skeletal muscles, dental tissues, etc. [22]. Although MSCs from each source have similar phenotypes and abilities to differentiate, they express distinct molecular profiles and differentiation potentials as the fingerprint suggestive of their origins [23,24]. Right after systemic infusion, MSCs mainly accumulate in the lungs, then gradually migrate to injured or inflamed tissues or to other organs such as the liver, spleen, kidneys, and bone marrow in non-injury models [14,25,26,27]. Therapeutic potentials of MSCs have been widely studied for cutaneous wound healing, muscle-bone-cartilage regeneration, Crohn’s disease, graft-versus-host disease, cardiovascular diseases, etc., with impressive outcomes and promising results [28]. Because MSCs can generate new neurons or other neural supporting cells, they are also an attractive target to develop treatment for neurodegenerative or neuroinflammatory disorders [29,30]. MSC-based therapy has been proven to be effective in ischemic stroke, neurodegenerative diseases, brain and spinal cord injuries [31], although in many cases, the exact mechanism of their therapeutic action remains to be elucidated.

### 2.2. MSCs as Vehicles for Targeted Delivery to the Brain

MSCs display an inherent and strong affinity to sites of injury, to which they display active migration from systemic circulation [32]. MSCs can effectively traverse the BBB to access injured sites located within the brain parenchyma. Despite limited crossing through the intact BBB, there is strong evidence of MSCs crossing it unassisted in certain conditions [33,34], particularly when tight junctions of this barrier are compromised due to physicochemical insults, inflammation, or the aging process [35,36]. As for brain ischemic stroke or trauma, intravenously administered MSCs can cross the BBB to integrate into damaged areas [33], while MSCs directly transplanted into brain parenchyma traffic through different cerebral areas [12] and to the infarcted regions [37]. As for malignant lesions, natural affinity to glioma has been demonstrated on neural stem cells (NSCs) [13], embryonic stem cells (ESCs) [38], and MSCs [39]. Homing of intracranially injected MSCs to brain tumors was reported in glioma [40] and medulloblastoma [41] models. MSCs and NSCs were also found to exhibit some anti-cancer effects against tumor development and progression [42,43]. While NSCs were first explored and their use has been translated into clinical trials, they face a serious issue of low cell numbers due to difficulty in obtaining them from human or from in vitro cultures [44].

Compared to other stem cells, MSCs have attracted a great deal of attention for therapy development, which can be attributed to several advantages. First, MSCs are abundant in various tissue types, easy to harvest from adult individuals, and can be expanded ex vivo in high quantities with minimal loss of function [45], supporting the use of patient-derived MSCs as autografts [46]. Second, MSCs have a relative immune-inert status [47,48]. This opens the door to MSC-derived allografts with less vigorous testing of immunocompatibility and allows development of off-the-shelf MSC-based products. Third, MSCs also pose a lower the risk of malignant transformation after in vivo transplantation because of their limited replication cycles compared to pluripotent cells. Finally, MSCs are tolerant of manipulations to improve their therapeutic abilities. For example, loading chemotherapeutic drugs into MSCs can significantly improve their anti-cancer effects from 15% to approximately 90% [49]. Fortunately, MSCs have some unique characteristics which make them an ideal vehicle for drugs. They can effectively internalize chemicals (e.g., paclitaxel, doxiburicin), and at the same time are resistant to those agents [50]. MSCs can also be genetically modified to produce therapeutic agents or to express specific targeting molecules which enhance their homing effect [51].

## 3. Current Status and Challenges of MSC-Based Therapies

### 3.1. Current Status of MSC-Based Therapies for Brain Tumors

For brain tumors, most preclinical studies exploit MSCs as a vehicle to deliver a wide range of therapeutic agents such as chemotherapeutic drugs [52], cytokines to stimulate immune responses against tumors [53] or to inhibit angiogenesis [54], molecules to induce apoptotic processes [55,56], enzymes for suicide gene therapy [57], oncolytic viruses [58], etc. The two most successful strategies in terms of advancement to clinical trials are those using allogeneic BM-MSCs to deliver oncolytic adenoviruses (NCT03896568) and deliver suicide genes (NCT04657315) to treat glioblastomas. It is also worth mentioning two other clinical trials using NSCs to apply the same two approaches for glioblastoma (NCT03072134 and NCT02015819). Readers are referred to other reviews for further knowledge on stem cell-based therapies for brain tumors [42,43,44,59]. However, using stem cells to treat diseases, especially cancer, remains a controversial subject [15,60]. Contrary to promising conclusions from many in vitro and in vivo investigations, a majority of stem cell-based therapy studies have shown disappointing performance when applied to human subjects with insignificant or modest outcomes [61]. This can be attributed to variations in multiple parameters in the selection of MSC sources, cultivation, and engineering processes, which imply the context-dependent characteristic of MSCs: those collected from different tissues and by different methods may acquire different properties.

### 3.2. Important Issues of MSC-Based Therapies

The challenges of MSC-based therapy are first situated in the techniques of handling the cells, with many variables that can greatly affect therapeutic properties of the final product. This highlights the essential need to standardize and strictly control current cell-based protocols. One may also expect the option of redirection towards other cell-free systems that are easier to standardize and control.

Cell-based strategies also face other challenges of biological efficacy and safety, which can be grouped into four different categories: (1) biostability in systemic circulation and homing ability, (2) interactions with the host immune system, (3) unexpected differentiation and potential tumorigenicity, and (4) other adverse effects such as infection transmission, embolisms caused by cell aggregations, etc. (Table 1). Despite their in vivo existence for up to 2.5 months in the xenograft model, MSCs have a poor rate of integration into target tissues [62,63]. The percentage of MSCs successfully engrafted turned out to be very low, probably because of their lodging in small vessels particularly in the lungs [64]. While autologous MSCs could persist for more than 200 days in the body, allogeneic cells rapidly disappeared from circulation, and in vivo rejection was observed [65]. Although MSCs are less likely to trigger immune responses compared to other stem cells, MSCs should be regarded as being able to ‘transiently escape’ the immune surveillance rather than being ‘immune-privileged’ [66].

A number of studies reported that naïve MSCs (i.e., natural, unmodified MSCs) can have pro-tumor effects [67,68], leading to the next question of their ability to promote cancer progression or even participate in de novo tumor formation [69]. So far, no studies using MSCs to treat glioblastoma have reported new tumor formation, but MSCs originating from different sources (e.g., AT-MSCs, UC-MSCs or BM-MSCs) did exhibit different behaviors of supporting or suppressing existing tumors [43]. Although no definite conclusions can yet be reached, one important point to emphasize is that results of MSCs supporting cancer growth were established in experiments using naïve MSCs. Most models using modified MSCs, on the other hand, have reported positive effects of tumor inhibition [51]. Modifications such as genetic engineering or drug loading can be beneficial in boosting therapeutic effects of MSCs, and also in negating their potential pro-tumor risks. Last, the rate of getting infections from allogeneic MSCs infusion was reported to be 29.5% [70], which again highlights the importance of cell source selection and rigorous testing of cell purity and biological properties.

Such serious but inherently weak points of cell-based therapies reflect a need to redesign current strategies and explore new ones. Stem cells were originally thought to execute their effects via effective access and engraftment into target tissues. However, results from various preclinical studies confirmed a correlation between the dosage of systemically administered MSCs and therapeutic effects, but found no correlation between the number of cells integrated into the site of disease and outcomes [71]. This implies that the observed benefits might not depend on, or be directly related to, the integration and differentiation of MSCs in the region of interest. Interestingly, most of the desired therapeutic effects of MSCs were found to reside in their secretome, including secretory molecules (growth factors, cytokines and other signaling molecules) and cell-derived extracellular vesicles (EVs) [17]. This suggests a “hit-and-run” mode of action that MSCs actually exert upon arrival at target tissues, hence opening the door to cell-free solutions based on MSCs. The rest of this review focuses on the biology and therapeutic potential of EVs originating from MSCs (MSC-EVs), available techniques to optimize them as nano-delivery systems, and the current status and challenges of research on EV-based therapies.

## 4. Extracellular Vesicle (EV) Biology

### 4.1. Classification and Formation

EVs are groups of nanoparticles coated by phospholipid-bilayer membranes which are present in various body fluids and released by all prokaryotic and eukaryotic cells through numerous cellular processes during both physiological and pathological conditions. EVs may contain various bioactive cargos, including DNA, RNA, proteins, lipids, and metabolites [72,73]. Based on the size, biogenesis pathway, membrane marker, specific function, and cargo contents, EVs are classified into three major subgroups: exosomes, microvesicles, and apoptotic bodies [74,75].

Exosomes have an average size of 30–150 nm in diameter, constructed through the inward budding of multi-vesicular bodies (MVBs) into the cytoplasm. Intraluminal vesicles (ILVs) are subsequently formed through the invagination of MVB membranes [76]. Specific cargos, such as nucleic acids, cytoplasmic proteins, lipids, and surface ligands from the plasma, are packed inside ILVs, which are either degraded or released as exosomes. Following MVB fusion with plasma membrane, exosomes are released into the extracellular space [77]. Compared to exosomes, microvesicles are larger with diameters of 200–1000 nm, and are generated from the direct outward budding and cleavage of the cell membrane. This budding mechanism allows membrane proteins and cargos to be encapsulated inside the microvesicles [75]. Apoptotic bodies are larger EVs with diameters of around 800–5000 nm and are generated from cell fragmentation and cytoskeletal breakdown during programmed cell death. Apoptotic bodies can carry shattered nuclear components or organelles and are destroyed by macrophages through phagocytosis [78].

### 4.2. Biologic Functions at Target Cells

Contrary to previous beliefs that they are just a means of cellular waste disposal, EVs, particularly exosomes, play a crucial role in intercellular communications by transferring unique information in the forms of their lipid membrane, surface molecules, or cargo compositions [72]. Exosomes bind to cell surface for further initiation of intracellular signaling pathways inside recipient cells, which can either internalize the exosome to mingle with endogenous ILVs, or keep the exosome bound to the cell surface [73]. The uptake process is mediated through different pathways, including receptor–ligand interactions, direct plasma membrane fusion, transmembrane signal transduction, endocytosis, phagocytosis, and macropinocytosis [79,80,81]. Such mechanisms serve one essential purpose of preventing exosomes from being degraded by lysosomal degradation pathways of the recipient cell. Then, the exosome can release its specific cargo into the cytoplasm, leading to alterations in the cell’s physiological or pathological status [82].

In general, EVs biologically mirror their cells of origin [83]. Molecular analyses revealed the resemblance of MSC-EVs to parent cells in terms of immunophenotype, lipid, and protein composition [84,85], and the horizontal transfer of RNA contents [86]. Since cells use their secreted EVs as a universal tool to transfer different signaling molecules to cross-talk with other cells, this mode of communication is not limited to the local microenvironment, but is also spread a very long distance via systemic circulation and biological fluids to reach remote organs [74,87]. In the fields of oncologic and neurologic studies, EVs were characterized to contain specific micro (mi)RNAs, proteins and other biological compounds that reflect the disease entity, grade, or progression status [88,89]. One blossoming branch of research is to collect and isolate endogenous EVs from body fluids such as plasma, urine, and saliva to extract information that can be useful for diagnostic or prognostic purposes [90,91]. Another therapy-oriented direction, which is also the main focus of this review, is to generate EVs ex vivo and then use them as therapeutics or as carriers of therapeutics for targeted delivery in vivo [92,93].

## 5. Potentials of MSC-EVs as a Cell-Free Platform of Therapeutic Delivery to the Brain

Given the well-studied natural capabilities of MSCs mentioned above, a branch of research on MSC-based therapies has diverged from using MSCs towards using MSC-EVs. The idea of employing secretomes instead of cells can avoid many critical issues of the cell-based approach, yet still make good use of beneficial abilities of parent cells to fight cancer and CNS diseases.

### 5.1. Advantageous Properties of MSC-EVs for Therapeutic Translation

#### 5.1.1. Intrinsic Ability of Homing to the Brain and Crossing the BBB

EVs can freely travel in and out the CNS in both physiological and pathological conditions [94]. EVs from several sources such as macrophages (Raw 264.7), dendritic cells, 293T cells, and MSCs have been employed to transfer exogenous biomolecules to the brain [95]. Studies on interactions between EVs and endothelial cells of the BBB have shed some light on how these vesicles cross this barrier. As a first-line protection of the brain parenchyma, the intact BBB has at least three defense mechanisms to restrict cellular and molecular transport: (1) tight junctions (TJs) between the BBB’s endothelial cells, which significantly hamper ions and other hydrophilic substances passing through the intercellular space, (2) membrane efflux pumps on the endothelial cell surface, and (3) extracellular and intracellular enzymes [95]. All of these components ensure that most exogenous agents can only penetrate the BBB at an extremely low rate, regardless of whether their way of traversing is paracellular (between the cells) or transcellular (across the cell).

In particular, the TJs block the paracellular pathway. Therefore, the integrity of these junctions being compromised can allow the infiltration of inflammatory cells and abnormal accumulation of fluids and substances, which are seen in many neurological diseases [96]. There are some specific situations where BBB disruption is induced by cancer-related activities occurring in the brain [97,98]. With glioblastomas, for instance, cancer cells form a bizarre vascularization by interacting with nearby blood vessels and breaking down the TJs to acquire resources for growth and invasion [99,100]. This leaky status of the BBB, also called the ‘blood-brain tumor barrier’ (BBTB), results in increased pore sizes and altered hydraulic conductivity [101]. Such a patchy structure and uneven permeability create ideal conditions for small molecules such as nanodrugs and exosomes to access local tumor tissues [100,102]. Except in pathological conditions, however, EVs have not been demonstrated to cross the intact BBB through paracellular pathways [94].

There is evidence that EV transport across the BBB mainly occurs via transcellular pathways, which can be receptor-mediated transcytosis (via specific ligand–receptor binding) or adsorptive-mediated transcytosis (via nonspecific interaction with membrane cationic proteins or peptides) [103]. This provides a foundation for the concept of active targeting: to modify the nanoparticle surface to richly express certain moieties that mediate or accelerate transcytosis processes at the BBB. Last but not least, a prerequisite for BBB crossing regardless of mechanisms is that EVs must be maintained in a high enough plasma concentration and a long enough duration to actually reach the brain. This represents the concept of passive targeting, in which the EVs’ superior homing to the brain is attributed to their effective escape of natural clearance [92].

#### 5.1.2. Good Biosafety and Bioavailability

In addition to comparable similarities in therapeutic potentials between MSCs and their derived EVs, EVs also possess a couple of pharmacokinetic advantages. EVs only induce a minimal immune response after introduction into the body due to their small size and basic structure. This stealth capability also comes from their endogenous surface composition with particular major histocompatibility (MHC) class I and class II expressions, making the EVs evasive to antigen-presenting cells [104]. However, EVs can still provoke a certain level of immune response, and should not be considered a non-immunogenic entity [105]. Systemically injected MSC-EVs are less likely to be trapped in the lung or liver compared to MSCs, resulting in lower clearance rate and higher half-life time in the circulation [106]. Distinct surface protein and lipid compositions of EVs allow them to evade immune recognition and escape filtration by the mononuclear phagocyte system (MPS) [85]. It is still largely unknown whether EVs exhibit other mechanisms to improve their retention in the blood.

### 5.2. Comparison of EV-Based Platforms with Other Delivery Approaches

#### 5.2.1. Cell-Based Versus Cell-Free Delivery Systems

Several studies compared the MSC-based versus MSC-EV-based therapies, showing similar outcomes and insignificant differences in therapeutic performance between the two approaches (reviewed in [66,107,108]). Visualization of the mouse brains after intranasal delivery of MSCs or MSC-EVs demonstrated that MSC-EVs produced much higher signals of integration into the brain parenchyma than MSCs, both on bioimaging with fluorescent labelling and on immunostaining [109]. In addition to the fact that MSC-EVs can mimic or surpass MSCs in several aspects, rationales behind the preference for a cell-free approach also come from several issues of the cell-based strategy itself. In vivo differentiation of MSCs at target tissues is extremely rare, partly due to that the time they reside in the tissues is notably short and widely varies from 48 h to 3 months [110]. Engrafted MSCs can still suffer from immune rejection, or the target tissue microenvironment is unfavorable for them to function as expected. EVs, on the other hand, can neither proliferate nor differentiate inside the human body, and thus raise fewer safety issues and much fewer ethical and legal concerns. The small size is also valuable in terms of sterilization, as EVs can become aseptic after filtration through a 0.22-µm membrane with minimal risk of carrying infections. MSC-EVs can be stored at −80 °C or even −20 °C with no influence on their biological function, while cryopreserved MSCs show deterioration in immunomodulatory and pro-regenerative capacity [111,112]. Last but not least, the process to generate EV-based products for clinical use is simpler, less costly, and easier to scale up compared to that of cell-based products (Table 1).

#### 5.2.2. EVs versus Synthetic Nanoparticles (NPs)

In addition to natural EVs produced by living cells, synthetic NPs are another type of cell-free system. The artificial production gives them a superior advantage of simple and easy-to-control production [113]. Polymeric and lipid-based NPs are commonly used in biomedical research. Since polymeric NPs were demonstrated to cross the BBB in vivo, some of them are being translated into therapy for brain tumors and other CNS diseases [113,114]. However, polymeric NPs are quickly cleared by the MPS, due to lack of specificity, along with serious drawbacks of high immunogenicity and toxicity which hinder their use in the clinic [115]. Lipid-based NPs, especially liposomes, are the more well-studied platform. Having a structure of a bilayer lipid membrane, liposomes encapsulate and protect many macromolecular drugs or nucleic acids from external factors. However, their non-endogenous origin still triggers massive entrapment in organs [114]. EV-based delivery, on the other hand, seems to be better in many aspects such as biocompatibility (less toxic and immunogenic), biostability (longer half-life times) and targeting ability (brain homing and tumor tropism) compared to artificial nanocarriers [116,117]. A comparison of the EV-based and liposome-based approaches concluded that EVs displayed better tumor tropism and higher therapeutic efficacy than liposomes [118]. EVs also had a 10-fold higher chance to be internalized by target cells compared to commercial liposomes of a similar size [119]. Using drug-loaded EVs was reported to have a 60% higher level of drug uptake by target cells compared to using free drug or drug-loaded liposomes [120]. EVs engineered to load miRNAs also displayed better therapeutic performance, although the cargo-loading capacity can be lower than that of synthetic NPs [93,121].

## 6. Engineering MSC-EVs to Become the Next-Generation Nanocarrier

Various strategies have been developed to increase the therapeutic performance of natural EVs. In many cases, parent cells need to be genetically or non-genetically modified to produce the desired EVs. Another option is to manipulate the EVs after being isolated from parent cells. Both approaches can serve different purposes: to efficiently pack anti-tumor agents into EVs, or to enhance tumor-targeting effects through the improvement of stability in systemic circulation, ability to cross the BBB, and tumor tropism.

### 6.1. Packaging Drugs and Genes into MSC-EVs

**Figure 1:**
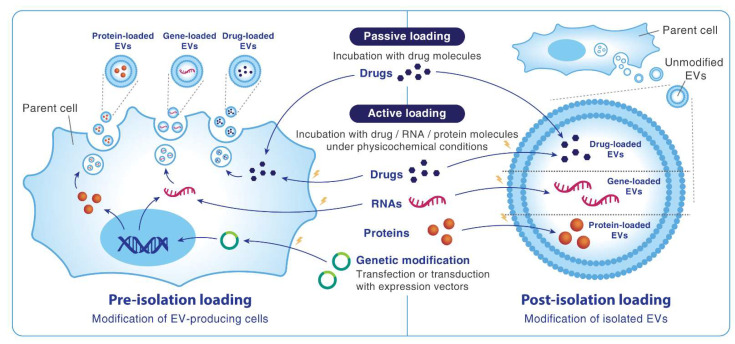
Methods of cargo loading into extracellular vesicles. EVs, extracellular vesicles.

There have been many studies reporting successful loading of different types of cargos into MSC-EVs [66,122,123]. Such cargos can be divided into three main categories: (1) small-molecule drugs (e.g., paxitacel, doxorubicin, curcumin), (2) proteins (e.g., TRAIL, CXCR4, GATA-4, neprilysin), and (3) nucleic acids, including messenger (m)RNAs, miRNAs, and siRNAs (Figure 1). Pre-isolation methods involve manipulation of parent cells during EV biogenesis to make their EVs contain specific molecules. The simplest form is to incubate cells with a drug for spontaneous cellular entrapment, leading to release of drug-carrying EVs. This process can be actively boosted with external factors such as electroporation. Another approach is to genetically modify progenitor cells via transfection or transduction with expression vectors, leading to endogenous synthesis and secretion of the desired product. In this way, pre-isolation methods allow one to harvest EVs that are already loaded with the cargo, eliminating the need to harshly interfere with the EV structure afterwards. However, this method faces the challenge of giving reproducible results because the mechanisms and levels of expression are difficult to control [66]. On the other hand, post-isolation techniques load cargos into the EVs after their isolation from cell culture. Simple co-incubation of EVs and the drug can be done, but additional interventions including electroporation, saponin permeabilization, freeze−thaw cycles, sonication, and extrusion are often required to achieve higher loading capacities. These techniques, however, can result in EV aggregation, complicate the isolation and purification processes, and may compromise some desired qualities of the final product [122].

A second way to classify cargo-packaging methods is to divide them into passive and active loading (Figure 1). Passive loading approaches are mostly applicable for small molecules such as chemotherapeutic drugs. Hydrophobic compounds have the highest loading efficacy as they can stably bind to the EV lipid bilayer. They can be either incubated with parent cells or directly mixed with the EVs. While the former strategy is inefficient due to dependence on the cell’s capacity to internalize and pack the drug into EVs, the latter strategy still has suboptimal loading capacity [107]. To overcome this challenge, electroporation can be applied on the parent cells or on the EVs to load large, charged and hydrophilic molecules such as miRNAs and siRNAs, which are inherently difficult to diffuse through biological membranes [95].

### 6.2. Improving the Functions of MSC-EVs

After being loaded with a therapeutic agent, EVs can require additional reinforcement to obtain satisfactory pharmacokinetics and targeting effects. Several technologies have emerged to meet those needs. Some studies exploited the concept of passive targeting to hinder the MPS-mediated recognition and phagocytosis of EVs using materials such as dextran sulfate [124] or polyethylene glycol to coat the EV surface [125]. Coatings, however, can potentially compromise the functional surface of EVs [92]. In a more direct way, active targeting aims to enhance homing capacity by decorating the EV surface with targeting molecules (mostly peptides or proteins) which preferentially bound to specific receptors or ligands on the target cell surface. Such molecules can be endogenously produced by parent cells via genetic modification. For example, transfection of dendritic cells or HEK293T cells with expression vectors encoding for rabies virus glycoprotein (RVG) peptides, which target the nicotinic acetylcholine receptors, have shown successful EV-based delivery to target neurons [126], increased cellular uptake [127], and increased EV accumulation in the brain parenchyma after IV administration [128]. This method can make use of components that are physiologically abundant on the EV surface such as Lamp2b [126,127,128] or glycosylphosphatidylinositol [129] to anchor and display the targeting molecules in a natural conformation. Alternatively, other studies produced exogenous peptides and later engrafted them on the EV surface via simple EV co-incubation with the peptides to target LDL receptors in the BBB [130], or via chemical linkage to target neuropilin-1 in gliomas [131] and α_v_β_3_ integrin in ischemic brain injuries [132]. Another approach takes advantage of the fact that natural EVs can express intrinsic moieties for tumor tropism, while synthetic liposomes can be easily manipulated to load cargos or acquire additional qualities. Then, the two types of particles are fused together to form a new form of ‘hybrid EV’, which has superior qualities of both EVs and liposomes [133,134]. Despite considerable research progress in EV functionalization, to our knowledge, there are no studies in brain tumors using these surface display techniques on MSC-EVs. Nevertheless, such available and well-studied methods can inspire future studies to make EV-based delivery systems more flexible, reprogrammable, and readily implemented into different research models.

## 7. Current Status of MSC-EV Therapeutic Applications in the Brain

Non-infectious neuropathologies of the brain consist of distinct entities such as neuroinflammatory and neurodegenerative disorders, ischemic and traumatic injuries, primary and secondary neoplasms, etc. Since EVs emerged as a potential therapeutic platform in this field, many preclinical models have achieved desirable EV distributions in the brain for effective drug delivery [94,95,103,116,117]. At present, more than 200 studies involving the use of EVs or exosomes are registered on clinicaltrials.gov (accessed on 1 September 2021). Many of those are observational, aiming to isolate EVs from patient-derived body fluids for diagnostic and prognostic purposes. There are four clinical trials on therapeutic application of MSC-EVs in brain disorders: acute ischemic stroke (NCT03384433), depression, anxiety, and dementias (NCT04202770), Alzheimer’s disease (NCT04388982), and craniofacial neuralgia (NCT04202783). Because using EVs to treat brain malignancies is still in its early infancy, insights into their application in other brain pathologies are fairly relevant and would be helpful for comprehensive and critical viewpoints on this topic. This section briefly introduces the current status of research using EVs from various sources in common brain diseases, then focuses on important preclinical studies using MSC-EVs in brain tumor models.

### 7.1. Non-Neoplastic Brain Diseases

Non-neoplastic brain disorders can result from neural injuries (e.g., cerebrovascular ischemic stroke, brain traumatic injury), degeneration process (e.g., Alzheimer’s disease, Parkinson’s disease, Huntington’s disease), autoimmune inflammatory response (e.g., multiple sclerosis) or other complex mechanisms (e.g., epilepsy). Recent encouraging results from MSC-based therapy in the field of degenerative and inflammatory brain disorders have driven further advancements towards using MSC secretomes [30,135]. From this aspect, unmodified MSC-EVs, i.e., without pre- or post-isolation modifications, can be utilized as the therapeutics themselves. In a mouse model with induced cerebrovascular ischemic injury, systemically administered EVs from human BM-MSCs resulted in enhanced angiogenesis, improved neurological impairment and long-term neuroprotection [136]. The same EV source was also applied in several rodent models of traumatic brain injury [137,138,139] and hypoxic-ischemic perinatal brain injury [140,141]. In addition, EVs isolated from some special subpopulations of MSCs were also proven effective in vivo, including stem cells from human exfoliated deciduous teeth (SCED) to target Parkinson’s diseases [142], and human periodontal ligament stem cells (PDLSC) for multiple sclerosis [143]. Other studies on multiple sclerosis [144,145], epilepsy [146] and autistic behaviors [109] also showed promising results from using unmodified MSC-EVs in rodent models.

In addition, engineered MSC-EVs have also been exploited to deliver RNAs or proteins with positive results, including miR-133b and miR-17-92 in rat models of stroke induced by middle cerebral artery occlusion (MCAO) [147,148], miR-124 in mice with cortical ischemia [149], miR-21 and neprilysin in Alzheimer’s disease [150,151]. Intranasal administration of MSC-EVs loaded with curcumin and functionalized with integrin-targeted surface peptides also reduced neuroinflammation in murine MCAO brains better than using curcumin alone [132]. For deeper knowledge of EV applications in non-cancerous neurologic disorders, readers are referred to other reviews [152,153].

### 7.2. Brain Tumors

#### 7.2.1. Studies Using MSC Secretomes

The past 10 years has witnessed the rapid development of EV-based research in the field of neuro-oncology, mostly focused on glioblastoma multiforme (GBM). Earlier findings about anti-tumor properties of MSC secretomes were done using conditioned medium (CM) of MSC cultures to treat cancer cells. There are reports on the inhibitory effects of UC-MSC CM on C6 glioma cell growth [154], inhibitory effects of AT-MSC and UC-MSC CM on U251 glioma cell proliferation, migration, and invasion [155], stimulatory effects of BM-MSC and UC-MSC CM on GBM stem-like cells’ response to temozolomide (TMZ) and cell-cycle arrest [156], and stimulatory effects of BM-MSC and WJ-MSC CM on U87 cell cell-cycle arrest [157]. A more recent study specifically used the exosomes isolated from CM of UC-MSCs, which expressed an inhibitory effect on U87 cell growth [158].

However, several studies reported some pro-tumor effects of various MSC sources, namely UC-MSC CM on glioma cancer stem cell proliferation [159], AT-MSC CM on C6 glioma cell migration and epithelial-to-mesenchymal-like transition [160], and human umbilical cord perivascular cell (HUCPVC, a specific population of WJ-MSCs) CM on U251 and SNB-19 glioma cells [161]. Furthermore, inconsistent effects of MSCs on the target cell’s malignant properties were observed within the same study, largely due to the differences in MSC origins. Del Fattore et al. demonstrated that BM-MSC- and UC-MSC-derived EVs could suppress U87 cell growth and induce apoptosis, but those derived from AT-MSCs supported cell proliferation and had no ability to facilitate apoptosis [162]. In line with this finding, studies by Onzi et al. using AT-MSC CM revealed no benefits in U87 cells proliferation, sphere-forming capacity, or sensitivity to TMZ, but increased cell migration (a tumor-supportive effect) [163] and blocked cell autophagy (a tumor-suppressive effect) [164]. These findings suggest that the type of MSCs used to produce EVs can be a crucial determinant of anti- or pro-tumor outcomes.

Some studies reported this ‘multi-faceted’ feature of MSCs originating from the same population. Li et al. highlighted the abilities of BM-MSCs CM to promote the migration and invasion but inhibit the proliferation of C6 cells [165]. Chistyakova et al. observed that CM collected from MSCs exerted no pro-proliferative effect on glioma cells, but those collected from MSCs co-cultured with glioma cells induced proliferation in other glioma cultures. Interestingly, this tumor-supportive effect was only observed when using culture medium harvested within the first 3–9 days of co-culture, while medium collected at later time points (15–21 days) had an inhibitory effect on glioma cell proliferation [166]. It is important to note that most of the conflicting reports were only conducted in vitro, and most of them used CM, which contained EVs and also a wide range of other complex molecules.

#### 7.2.2. Studies Using Engineered MSC-EVs

Another trend of MSC-EV research focuses on using engineered MSC-EVs to deliver different anti-cancer agents to exert effects on target cells. Findings about EV-mediated transfer of miRNAs from MSCs to glioma cells [167] inspired the idea of translating this biologic process into gene therapies (Table 2). In a pioneer study by Katakowski et al., BM-MSCs were transfected with miR-146 expression vector to produce MSC-EVs with a 7.3-fold increase in the miRNA-146 content, which showed an inhibitory effect on rat gliosarcoma cell (9 L) growth. An in vivo intra-tumoral injection of the same EVs into rat brains bearing 9 L gliosarcomas also significantly reduced the tumor volume [168]. Munoz et al. reported the important role of miR-9 in GBM cells’ resistance to TMZ, hence aiming to reverse the resistant status by using anti-miRNAs loaded into MSCs. In particular, MSCs were transfected with synthetic Cy5-labeled anti-miR-9 and then co-cultured with GMB cells, resulting in decreased expression of drug efflux transporters and recovered sensitivity to TMZ. Importantly, EVs isolated from MSCs and added to GBM cells also produced the same re-sensitization effects. The authors found that the transfer of anti-miR-9 between two cell types can occur via both cell–cell gap junctions and exchange of microvesicles, with the latter being the more prominent pathway [169]. The same pre-isolation method to load miRNAs into MSC-EVs was reported by Lee et al. MSC-based delivery of Cy3-labeled miR-124 and miR-145 was also found to be mediated via both gap junctions and exosomes, and could inhibit migration of U87 cells and reduce the self-renewal ability of glioma stem cells [170]. Findings from Lee’s work on miR-124 were supported by another in vitro study using a different EV source of WJ-MSCs, reporting anti-cancer effects of miR-124-loaded EVs on glioma cells proliferation, migration, and sensitivity to TMZ [171].

Regarding such potential in vitro discoveries on miRNAs, Lang et al. was the first group to perform an animal study to challenge the concept of systemically injecting MSC-EVs loaded with miRNA to treat brain tumors [172]. Among eight potential miRNAs with anti-glioma properties, miR-124a was the most effective one and was stably overexpressed in BM-MSCs using a lentiviral system. Primary glioma stem cells (GSCs) treated with MSC-EVs carrying the microRNA (Exo-miR124) exhibited reduced proliferation and clonogenicity in vitro. Mice with orthotopic implantation of GSCs were intraperitoneally administered Exo-miR124 every 2 days, and 50% of animals were still alive at 110 days with complete regression from pathological analysis of surviving mice [172]. Following this work, many studies on GBM also adopted similar delivery strategies to validate the therapeutic potential of different miRNAs including miR-584 [173], miR-133b [174], miR-199a [175], miR-375 [176], miR-512-5p [177], and miR-29a-3p [178]. All studies chose to genetically engineer the MSCs, either through direct transfection with synthetic miRNAs [173,174,175] or using lentiviral transduction to stably and endogenously express the miRNAs [176,177,178], and then isolate the secreted EVs. Xenograft nude mice were the model of choice, with GBM cells inoculated into the brain via intracranial [177,178] or subcutaneous [173,174,175,176] injections (Table 2).

One ingenious approach is EV-based adaptation of suicide gene therapy from cell-based systems. In this kind of therapy, cancer cells are delivered with the gene encoding an enzyme that converts a non-toxic drug (prodrug) into its toxic form. Tumor cells that receive and express the enzyme are killed upon systemic administration of the prodrug. Non-expressing cells located nearby are also affected and die from the ‘by-stander effect’ [53,59]. This strategy has an additional advantage of keeping the stem cell populations in control because they are also eradicated, minimizing the risk of unwanted differentiation. MSCs have been utilized as a cellular vehicle to deliver two enzyme–prodrug systems to treat GBM, namely thymidine kinase gene from herpes simplex virus (HSV-TK) coupled with ganciclovir [183], and cytosine deaminase (CD) gene combined with 5-fluorocytosine [184]. Recently, there are two reports testing the use of MSC-EVs instead of MSCs to deliver suicide genes [179,180]. The rationale behind this replacement is that the observed effectiveness of MSC-based suicide therapy in brain tumors mainly comes from EVs secreted by the infused MSCs, while a majority of those cells will not reach the brain [185]. Both studies used retroviruses to transduce MSCs with expression vector encoded for yeast-derived CD with uracil phosphoribosyl transferase [179] or HSV-TK [180], then the exosomes were isolated and were proven to carry the enzyme transcripts. Glioma cell lines were treated with CM containing these EVs in combination with the prodrug, resulting in reduced cell growth compared to those treated with the prodrug only [179,180]. However, there are no in vivo experiments to support the efficiency of this strategy.

Targeted delivery of tumor necrosis factor-related apoptosis-inducing ligands (TRAILs) is another feasible application of MSCs in glioblastoma [56] and medulloblastoma [55]. These special molecules can bind to native receptors on the target cell surface to effectively activate apoptotic pathways and cause cell death. Since recombinant TRAILs have low therapeutic performance due to poor bioavailability and high toxicity [186], their encapsulation into delivery vehicle is necessary. MSCs also display an exceptional resistance to TRAILs, making them ideal biofactories to produce and carry such molecules [187]. In addition, the C-X-C chemokine receptor type 4 (CXCR4) is highly expressed by MSCs to regulate their affinity to immune cells and recruitment to injured sites [188]. MSCs overexpressed with CXCR4 to improve brain engraftment were also reported [189]. Recently, a combination of these two strategies in an EV-based system was performed by Liu et al., using an in vivo model of breast cancer cells metastasizing to the brain. BM-MSCs were transduced for co-overexpression of CXCR4 (as the enhanced targeting moiety) and TRAIL (as the therapeutic agent) to produce multi-functional exosomes, which was coupled with carboplatin treatment for synergistic anti-cancer effects [182].

One novel study was recently reported by Parker et al., being the first to target glioblastoma with siRNA-loaded EVs derived from MSCs [181]. Aberrant fusion of the two genes FCFR3 and TACC3 (F3-T3) was previously detected as an oncogenic driver in 3–8% of glioblastomas [190,191], and thus being an attractive therapeutic target. A specific siRNA (iF3T3) was designed to bind to the fusion breakpoint in order to minimize off-targeting influence on normal cells, and was packed into EVs derived from UC-MSCs using electroporation. Effective in vitro knock-down of F3-T3 was achieved with reduced viability of cancer cells [181]. However, no in vivo data were available in this report, and future investigation is needed to prove the applicability of this strategy.

## 8. Challenges in Applying MSC-EVs to Treat Brain Tumors

While EVs have great potential to become the next-generation system of drug delivery to the brain, unfortunately, their use has been strictly limited due to controversial results as mentioned above. This section presents current challenges with MSC-EV application, which can be separated into factors related to EV generation before use, and factors related to application of EVs in recipient subjects (Table 1).

### 8.1. Challenges of EV Production and Characterization

Secretion and composition of EVs are heavily dependent on the origin and senescence of their parent cells, as well as on factors present during their biogenesis, such as cultivation conditions and biochemical stimuli, processes of extracting the secreted vesicles from culture media, and subsequent handlings such as isolation, purification, formulation and storage of the final products. Variations in any of these steps can result in slightly to greatly different products, which may be an explanation for the variability across laboratories and study outcomes.

#### 8.1.1. Cell Types of Origin and Cell Senescence

It was reported that EVs derived from different MSC sources can exhibit different compositions and properties [192]. One study showed the advantageous profile of BM-MSCs regarding the EV production capacity [193], while other reports revealed the higher potential of MSCs derived from adipose or cardiac tissue in angiogenesis [194,195]. The mechanism of action seems to be an insightful criterion to choose the best MSC sources. When pro-angiogenesis or anti-inflammation is needed, for example, AT-MSCs and UC-MSCs may be preferred to BM-MSCs [196], but when pro-apoptotic and anti-proliferative qualities are wanted, BM-MSCs and UC-MSCs can be better options compared to AT-MSCs [162]. Another critical aspect is that parent cells, either primary cells from a donor or established in vitro cell lines, exhibit cell senescence over time. This aging can be both senescence from replicative cell cycles in cultivation and senescence associated with the age of the donor [197]. Some studies indicated that although aged MSCs can secrete more EVs than young MSCs, their EVs were also abnormally changed in miRNA content [198] and lost the protective effect in acute lung injury model [199] or the rejuvenating effect on hematopoietic cells [200]. To ensure an infinite supply of EVs, immortalized MSCs and MSC-like cells can be used to maintain cell properties during expansion, and also to avoid issues from primary cells [201].

#### 8.1.2. Cell Culture Systems and EV Isolation Methods

In good manufacturing practice (GMP)-based protocols, defined xeno-free and EV-free culture media are used to eliminate contamination or impurities. They were reported to have minimal effects on cell viability, and even be able to increase EV secretions [202,203]. To further boost EV yields, cell surface-to-volume ratio was increased via 3D-culture systems such as multilayered cell culture flasks, spheroidal aggregates, hollow-fiber bioreactors, and stirred-tank bioreactors. The last two methods have more potential due to being scalable, GMP-compliant, and incorporated in a closed system [196]. The next crucial step in the EV production workflow is to isolate EVs from the culture medium. There are many different methods of EV isolation being reviewed elsewhere [204], each of which has its own pros and cons in terms of EV yield, purity, cost, and scalability for industrial production. In the final steps, product formulation and methods of long-term storage are involved. In addition to traditional cryopreservation, EVs can be lyophilized and stored at room temperature [205], which is more convenient and economical in terms of transport, storage, and flexibility for off-the-shelf use than cell-based products.

One of the main challenges of EV-based therapeutic translation is finding an EV generation and characterization strategy that is GMP-compatible, measurable for quality control, reproducible for the desired effects, cost-effective and able to be scaled up [196]. Although several GMP-grade protocols were proposed [112,206,207], there is still no exclusive or standard approach for a universal application across studies. Furthermore, because many parameters can affect the resultant EV characteristics, every EV batch should include all detailed information on the production process.

### 8.2. Challenges Related to In Vivo Application of EVs

After production and characterization, the next issues that EV-based cancer research needs to address are related to applications in experimental models and the human body. Some promising results were incapable of being replicated due to insufficient details about the model, such as pathological subtypes and disease progression, target tumor or tumor-related cell types, how and when the treatment was applied, etc. Moreover, a proportion of in vivo studies utilized human-derived EVs in animal models to prove their hypothesis (Table 2). While there is no study evaluating the effects of xenogeneic EVs in organisms, it is certain that EVs from different species differ. Findings and conclusions drawn from such studies, therefore, should be viewed with caution [196]. The safety and performance of EVs in vivo are other factors for consideration, including the optimal administration route, biodistribution and stability in circulation, risk of tumor progression, and specific EV behaviors in the tumor microenvironment (TME).

#### 8.2.1. Pro-Tumor Effects

Can MSC-EVs switch their function towards supporting cancer cells? In addition to the mentioned anti-glioma versus pro-glioma dual effects of MSC-EVs, these multi-faceted behaviors were also reported in other types of cancer [123,208,209]. Due to this controversy, MSC-EVs are considered a ‘double-edged sword’ in cancer therapy [123]. In a study by Roccaro et al., EVs isolated from BM-MSCs of multiple myeloma (MM) patients promoted MM progression, while EVs isolated from BM-MSCs of healthy individuals had an inhibitory effect. The explanation was attributed to significant differences in miR-15a amounts carried by those EVs [210]. Another study reported that Transwell co-culture of MSCs and breast cancer cells distinctively altered the miRNA profiles of MSC-EVs, which promoted cancer cell quiescence and drug resistance. This dormancy, however, could be overturned using MSC-EVs loaded with anti-miRNAs to treat cancer cells [211].

It can be inferred from those findings that after interaction with cancer cells, MSCs seem to adapt their secretory profiles to become favorable, adverse, or ambivalent in terms of therapeutic purposes. Nevertheless, tumor promotion is proposed to occur via at least three possible mechanisms. First, mediators secreted by MSCs can participate in more than one regulatory pathway, each of which can be either pro-tumor or anti-tumor and thus can go against or cancel each other out. Second, the unexpected effect can be an indirect consequence of MSC-related impacts on other TME components such as blood vessels or the extracellular matrix, which in turn can play a supportive role for tumor cells. Finally, MSC-derived secretomes can recruit different immune cells or progenitor cells to the TME and exhibit a complex interactive network with existing components in the TME [22]. This emphasizes the need for both qualitative and quantitative characterization of MSC-EVs, especially their protein and nucleic acid signatures. More future work is needed to answer such questions about MSC-EVs’ complex behaviors in the TME.

#### 8.2.2. Pharmacokinetics and Routes of Administration

After intravenous (IV) infusion, how long EVs are maintained with high concentrations in the circulation is extremely important, because this time needs to be sufficiently long for EVs to travel and reach the brain parenchyma. Unfortunately, this period was found to be only 2 min, with less than 5% of systemically administered EVs present in the serum 5 min after injection [212,213]. EVs could be found 48 h post-infusion, but mainly accumulated in the lungs, liver, spleen, and pancreas due to uptake activity by the MPS [214,215]. In addition to systemic routes, other preferred approaches are to use local routes such as intranasal and intrathecal administrations for a more direct delivery to the CNS. Each of these was demonstrated to give better access to the brain but with different efficacies [216,217]. A comparison of MSC-EVs injected via each route (IV, intranasal, intrathecal, and intraperitoneal) in mice concluded that intranasal and intrathecal routes were more effective [218]. As expected, the intrathecal route, i.e., to directly inject EVs into the cerebrospinal fluid through the subarachnoid space, was the most effective, but it is also invasive with potential complications. This is not suitable for conditions that may require repeated dosing, such as brain malignancies. Therefore, the less invasive intranasal route, in which EVs were proposed to travel through the olfactory and trigeminal nerve pathways to reach the brain, seems to be the most promising method. In an epilepsy mouse model, EVs derived from BM-MSCs reached the hippocampus within 6 h after intranasal administration [146]. MSC-EVs also crossed the BBB more efficiently when applied via intranasal route compared to systemic route [109]. In general, an effective route can improve EV biodistribution and reduce the dose needed to achieve the same therapeutic effect in the brain.

## 9. Conclusions and Future Directions

Brain tumors remain a clinical challenge due to limited screening methods, lack of choice in interventions, and aggressiveness of some malignant forms. Despite vigorous treatments, many patients still experience poor outcomes due to iatrogenic side effects, therapeutic resistance, and tumor recurrence. Recent advances in omics studies provided insights into the molecular heterogeneity of CNS tumors, opening a new era of precision medicine for many tumors such as high-grade gliomas and medulloblastomas [219,220]. However, the prognosis of highly malignant brain tumors has only witnessed slight improvements in the past decade [1,2], despite preclinical development of multi-modal combinations, adjuvant drugs, and potential targets for precision therapy. Since any kind of therapeutic candidate for brain tumors can fail in vivo due to consequences of untargeted distribution, the top priority is designing a platform to deliver such agents to tumor sites for effective exertion of anti-tumor effects.

MSCs are in the spotlight due to their unique properties of fighting neurological and oncological diseases. Research using MSCs to carry anti-glioma agents has advanced to clinical trials, although this cell-based approach has raised many safety concerns in cancer therapy given the unknowns of their in vivo multiple actions [221]. EVs have also attracted attention for their ability to travel freely across the BBB, making them an ideal ‘window’ into the CNS [19]. EVs derived from MSCs not only exhibit valuable properties of their parent cells, but also inherently bypass many critical issues related to the living cells. EVs are also more tolerant of technical modifications to enhance or to obtain additional qualities, making them a good candidate to realize the shift from cell-based to cell-free therapy.

Concerning the controversy of the tumor-supportive behaviors of MSC-EVs, it should be noted that most reports on pro-tumor outcomes used unmodified MSC-EVs. One plausible explanation for the varied observations across studies is the context-dependent manner of MSC-EVs, which might switch between supporting and suppressing cancer cells according to variations in multiple parameters in EV production or in vivo applications. While achieving a standardized protocol can take a very long time and lots of effort, it would be reasonable to exploit MSC-EVs as a vehicle to deliver therapeutic agents, rather than using natural MSC-EVs as the therapeutics themselves. However, the heterogeneity in brain tumor [222] may impact targeting therapies. Due to limited package size, some special therapeutic agents may not be able to carry by MSC-EVs. Further studies will be necessary to address the issues of transporting macromolecules by MSC-EVs.

More basic studies are needed to investigate the actions of EVs at the BBB and in the TME. Novel methods to enhance homing to the brain and tropism to tumors would also be useful as the minimal EV dosages can be lowered to reduce off-target effects. There are other potential research directions waiting to be explored in this field, such as (1) continuing to evaluate the feasibility and efficacy of other anti-cancer agents which have been applied in cell-based systems, and testing with different tumor models and administration routes, (2) co-delivering multiple mechanisms in one form of EVs to boost therapeutic efficacies, (3) discovering the synergistic or re-sensitizing modes of interactions among EV treatment, chemotherapeutic drugs, surgery, and radiotherapy, (4) addressing novel targets of gliomas, and (5) verifying the MSC-EV delivery systems in other types of CNS malignancy. The future of cell-free targeted therapy for brain tumors is getting closer, yet warrants more efforts to validate the applicability in the human body and resolve critical challenges with this approach.

## Figures and Tables

**Table 1:** Comparison of important issues and challenges of mesenchymal stem cell (MSC)-based and MSC-derived extracellular vesicle (MSC-EV)-based approaches in targeting brain tumors.

	MSCs	MSC-EVs
**Therapeutic effects**	Ineffective engraftment in target tissues including the brain	Better BBB penetration and higher accumulation in brain parenchyma
	Limited options to improve therapeutic effects	More adaptable to a wider range of techniques
**In vivo application-related issues**	Risks of uncontrolled differentiation, immunotoxicity, infection, and embolisms	Better safety profiles
**Technical issues**	Limited capacity of ex vivo expansion and large-scale production	Higher yield and easier to scale up
	Complicated processes of final product formation, long-term storage and clinical usage	Less complicated processes
**Common challenges of both approaches**	Poor targeting efficiency due to entrapment in organs after systemic injection Potential of pro-tumor effects Lack of standardized protocols for production and characterization	

**Table 2:** Studies on brain tumors using engineered mesenchymal stem cell-derived extracellular vesicles (MSC-EVs) to deliver therapeutic cargos in vitro and in vivo.

Cargo Type	Condition	Study Model	Cargo	Cargo-Loading Method	EV Cell Source	Ref.
**miRNA**	Rat brain tumor	in vivo, rat, intracranial xenograft (9L)	miR-146b	Pre-isolation (expression vector transfection using electroporation)	MSCs	[168]
	Glioblastoma	in vitro, human chemoresistant GBM (T98G, U87)	anti-miR-9	Pre-isolation (synthetic miRNA transfection)	BM-MSCs	[169]
	Glioblastoma	in vitro, human GBM (U87) and primary GSC	miR-124, miR-145	Pre-isolation (synthetic miRNA transfection)	BM-MSCs	[170]
	Glioblastoma	in vitro, human GBM (U87)	miR-124	Pre-isolation (synthetic miRNA transfection)	human WJ-MSCs	[171]
	Glioblastoma	in vivo, mouse, intracranial xenograft (primary GSC)	miR-124a	Pre-isolation (lentiviral vector transduction)	human BM-MSCs	[172]
	Glioblastoma	in vivo, mouse, subcutaneous xenograft (U87)	miR-584	Pre-isolation (synthetic miRNA transfection)	human MSCs	[173]
	Glioblastoma	in vivo, mouse, subcutaneous xenograft (U87)	miR-133b	Pre-isolation (synthetic miRNA transfection)	mouse MSCs	[174]
	Glioblastoma	in vivo, mouse, subcutaneous xenograft (U251)	miR-199a	Pre-isolation (synthetic miRNA transfection)	human MSCs	[175]
	Glioblastoma	in vivo, mouse, subcutaneous xenograft	miR-375	Pre-isolation (lentiviral vector transduction)	human MSCs	[176]
	Glioblastoma	in vivo, mouse, intracranial xenograft (U87)	miR-512-5p	Pre-isolation (lentiviral vector transduction)	human BM-MSCs	[177]
	Glioblastoma	in vivo, mouse, intracranial xenograft (U87)	miR-29a-3p	Pre-isolation (lentiviral vector transduction)	human BM-MSCs	[178]
**mRNA**	Glioblastoma	in vitro, rat gliosarcoma (C6)	yCD:UPRT	Pre-isolation (lentiviral vector transduction)	human MSCs	[179]
	Glioblastoma	in vitro, human GBM (U118, 8MG-BA)	HSV-TK	Pre-isolation (lentiviral vector transduction)	human MSCs	[180]
**siRNA**	Glioblastoma	in vitro, human GBM (U87, SNB19)	siRNA to the F3-T3 breakpoint	Post-isolation (synthetic siRNA incorporation using electroporation)	human UC-MSCs	[181]
**Protein**	Brain metastasis	in vivo, mouse, intracranial xenograft (MDA-MB-231Br)	TRAIL and CXCR4	Pre-isolation (lentiviral vector transduction)	rat BM-MSCs	[182]

Abbreviations: miRNA, microRNA; mRNA, messenger RNA; siRNA, small-interfering RNA; GBM, glioblastoma multiforme; NPC, neural progenitor cell; GSC, glioma stem cell; MSC, mesenchymal stem cell; BM-, bone marrow-derived; WJ-, Wharton’s jelly-derived; UC-, umbilical cord-derived; yCD:UPRT, yeast cytosine deaminase with uracil phosphoribosyl transferase; HSV-TK, thymidine kinase gene from herpes simplex virus; TRAIL, tumor necrosis factor-related apoptosis-inducing ligand; CXCR4, C-X-C chemokine receptor type 4.
